# Ablation of Vitamin D Signaling Compromises Cerebrovascular Adaptation to Carotid Artery Occlusion in Mice

**DOI:** 10.3390/cells9061457

**Published:** 2020-06-12

**Authors:** Éva Pál, László Hricisák, Ágnes Lékai, Dorina Nagy, Ágnes Fülöp, Reinhold G. Erben, Szabolcs Várbíró, Péter Sándor, Zoltán Benyó

**Affiliations:** 1 Institute of Translational Medicine, Semmelweis University, 1094 Budapest, Hungary; hricisak.laszlo@med.semmelweis-univ.hu (L.H.); agneslekai@gmail.com (Á.L.); dorinanagy1011@gmail.com (D.N.); fulop.agnes@med.semmelweis-univ.hu (Á.F.); sandor.peter@med.semmelweis-univ.hu (P.S.); 2 Department of Biomedical Sciences, University of Veterinary Medicine Vienna, 1210 Vienna, Austria; Reinhold.Erben@vetmeduni.ac.at; 3 Department of Obstetrics and Gynecology, Semmelweis University, 1082 Budapest, Hungary; varbiro.szabolcs@med.semmelweis-univ.hu

**Keywords:** carotid artery occlusion, pial collateral circulation, vitamin D receptor deficiency, cerebrovascular dysregulation, atherosclerosis

## Abstract

Vitamin D insufficiency has been associated with increased incidence and severity of cerebrovascular disorders. We analyzed the impact of impaired vitamin D signaling on the anatomical and functional aspects of cerebrovascular adaptation to unilateral carotid artery occlusion (CAO), a common consequence of atherosclerosis and cause of ischemic stroke. Cerebrocortical blood flow (CoBF) showed a significantly increased drop and delayed recovery after CAO in mice carrying a functionally inactive vitamin D receptor (VDR) with the most sustained perfusion deficit in the temporal cortex. To identify the cause(s) for this altered adaptation, the extent of compensatory blood flow increase in the contralateral carotid artery and the morphology of pial collaterals between the anterior and middle cerebral arteries were determined. Whereas VDR deficiency had no significant influence on the contralateral carotid arterial blood flow increase, it was associated with decreased number and increased tortuosity of pial anastomoses resulting in unfavorable changes of the intracranial collateral circulation. These results indicate that VDR deficiency compromises the cerebrovascular adaptation to CAO with the most sustained consequences in the temporal cortex. The dysregulation can be attributed to the altered development and function of pial collateral circulation whereas extracranial vessels may not be impaired.

## 1. Introduction

Vitamin D is a key regulator of cellular functions and its deficiency has been implicated recently in the development of several diseases including cerebrovascular disorders [1,2,3]. There are many causes of vitamin D deficiency including reduced synthesis in the skin, decreased bioavailability, decreased synthesis of the active form of vitamin D—1,25-dihydroxyvitamin D—or heritable disorders, such as the hereditary vitamin D resistant rickets (HVDRR) [1]. HVDRR is caused by mutations in the nuclear vitamin D receptor (VDR), which results in generalized resistance to the effects of 1,25-dihydroxyvitamin D [4]. The active form of vitamin D regulates the expression of numerous genes—including those involved in vascular homeostasis—in a VDR-mediated manner [2]; therefore, HVDRR causes the loss of the beneficial biological actions of vitamin D, including those in the vascular system [4].

Although Mendelian randomization analyses have failed to provide evidence for a causal association between vitamin D levels and cerebrovascular diseases [5,6,7], observational studies imply a strong link between vitamin D deficiency and risk [8,9,10,11], as well as severity, of ischemic stroke [12]. Ischemic stroke is one of the leading causes of death; up to 5% of stroke cases are due to occlusion of the carotid arteries [13,14]. After unilateral carotid artery occlusion (CAO) the intracranial collateral circulation supplied primarily by the contralateral carotid artery represents the first line of defense against cerebral ischemia. The intracranial collateral circulation involves both large vessels of the Willis circle and smaller pial anastomoses between the terminal branches of the anterior, middle, and posterior cerebral arteries [15,16,17,18]. As vitamin D deficiency has been reported recently to cause marked morphological and functional alterations of cerebral arteries [19,20], we hypothesize that the collateral pathways might also be compromised resulting in an insufficient adaptation of the cerebrocortical circulation to CAO. Therefore, the aims of the present study are 1) to determine the consequences of impaired vitamin D signaling in the cerebrovascular adaptation to CAO and 2) identify the alteration(s) of the cerebral vasculature responsible for the diminished adaptation in mice carrying a functionally inactive VDR.

## 2. Materials and Methods

### 2.1. Animals

The experiments were performed on adult (median and interquartile range of age: 103 (98–122) days) male mice carrying a mutant, functionally inactive vitamin D receptor (VDR^Δ/Δ^) and their wild-type (WT) littermates on C57BL/6 genetic background [21]. The WT and VDR^Δ/Δ^ mice were bred by intercrossing heterozygous animals. Animals were housed at constant temperature with a 12/12 h light/dark cycle under specific pathogen-free conditions, and they had ad-libitum access to food and water. The breeding mice and their offspring were fed lifelong with chow enriched with calcium, phosphate, and lactose (S8852-S010, SM Rescue Diet VDR KO, ssniff Spezialdiäten GmbH, Soest, Germany) to normalize calcium homeostasis [21]. The experiments were carried out according to the guidelines of the Hungarian Law of Animal Protection (XXVIII/1998) and were reported in compliance with the ARRIVE (Animal Research: Reporting in Vivo Experiments) guidelines. All procedures were approved by the National Scientific Ethical Committee on Animal Experimentation (PEI/001/2706-13/2014, approval date: 17 December 2014).

### 2.2. In Vivo Experiments

In vivo laser-speckle imaging was used to determine the cerebrocortical blood flow (CoBF) changes following unilateral common carotid artery occlusion in 10 WT and VDR^Δ/Δ^ mice each. In another set of experiments, the blood flow changes of the contralateral common carotid artery were measured after unilateral CAO in 6 WT and VDR^Δ/Δ^ mice each. In both experiments, the mice were anesthetized with isoflurane (2%) during femoral artery cannulation and with intraperitoneally applied ketamine (100 μg/g body weight, Calypsol; Gedeon Richter, Budapest, Hungary) and xylazine (10 μg/g body weight, CP-Xylazine; CP-Pharma, Burgdorf, Germany) throughout the rest of the experiment. The depth of anesthesia was frequently tested by checking the plantar nociception or corneal reflex, and additional anesthetic was administered if necessary. Following the cannulation of the left femoral artery, the trachea was exposed in order to improve the breathing of the mice through an intratracheal cannula. Subsequently, the carotid sheath was gently dissected with particular care to preserve the vagus nerve [17]. For the later induction of CAO, a ligature with a loose knot was placed around the left common carotid artery. For monitoring the contralateral carotid arterial blood flow increase, both external carotid arteries were ligated, and flow probes were placed around both common carotid arteries. All surgical procedures were performed under a stereomicroscope (Wild M3Z, Heerbrugg, Switzerland), and the body temperature of mice was maintained between 37 and 38 °C throughout the experiments by using a heating pad, controlled by a rectal thermometer. The systemic arterial pressure was measured continuously using the left femoral artery cannula, whereas the oxygen saturation, heart rate, and respiratory rate were determined using a pulse oximeter (MouseOx Plus, Starr Life Sciences Corp., Oakmont, PA, USA) on the depilated right hindlimb. At the end of the experiments, the following parameters of the mice were determined: body weight, tibial length, heart weight, heart weight/body weight ratio, left ventricle weight, and brain weight.

### 2.3. Measurement of Cerebrocortical Blood Flow Using Laser-Speckle Imaging

**Figure 1:**
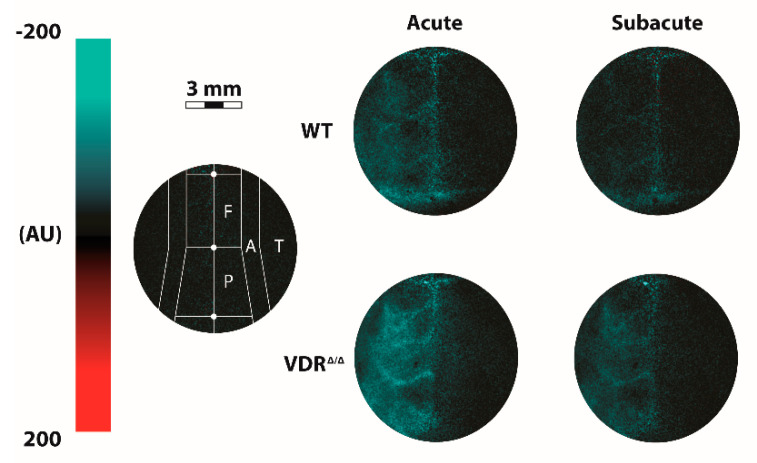
Localization of the regions of interest for cerebrocortical blood flow (CoBF) measurements and the CoBF reductions in the acute and subacute phase of adaptation after left carotid artery occlusion (CAO). The decrease in CoBF ipsilateral to CAO was more pronounced in the vitamin D receptor (VDR^Δ/Δ^) mice as compared to wild-type (WT) animals with the most sustained reductions in the temporal cortex (indicated by blue color). The first 30 s after CAO was considered as the acute phase, and the following 270 s was the subacute phase of adaptation. AU: arbitrary units, F: frontal cortex, P: parietal cortex, A: zone of pial anastomoses, T: temporal cortex.

The cerebrocortical blood flow was measured using laser-speckle imaging (PeriCam PSI; Perimed, Järfälla, Stockholm, Sweden). After the surgery, the head of the mouse was secured in a stereotaxic head holder, and the skull was exposed by retracting the scalp following a midline incision. First, atipamezole (1 μg/g ip.; Sigma-Aldrich, St. Louis, MO, USA) was administered as an antidote to xylazine, to reverse xylazine’s α-2-agonistic effects and in this way to ensure a stable blood pressure throughout the experiment [17]. We waited until atipamezole exerted its effect (ca. 10 min) [17], then 10 min were allowed to acquire baseline data of CoBF and blood pressure. Thereafter, the left common carotid artery was occluded by tightening the loose knot around the vessel [17]. The average baseline CoBF for 1 min preceding CAO was determined as a reference value (100%), and changes in CoBF until 5 min after CAO were recorded and expressed as a percentage of the reference CoBF. The area between the CoBF curves of the hemispheres ipsilateral and contralateral to CAO was determined for each animal. The first 30 s after CAO was considered as the acute phase, and the following 270 s as the subacute phase of adaptation. The CoBF changes were measured in four predefined and standardized cortical regions of interest (ROI): frontal, parietal, temporal cortices, and the zone of pial anastomoses of both hemispheres. The selection criteria of ROIs were reported previously [17] and are shown in Figure 1. Data analysis was performed by investigators who were blinded to the genotype of the mice.

At the end of each experiment, the femoral artery cannula was used for arterial blood sampling to determine arterial blood gas tensions, acid/base parameters, and plasma ion concentrations (Ca^2+^, Na^+^, K^+^, Cl^−^). If arterial O_2_ saturation was < 90% or CO_2_ tension was out of the range of 25–55 mmHg or the systemic mean arterial blood pressure was out of the range of 70–120 mmHg, the experiment was excluded from the evaluation. Complete occlusion of the common carotid artery was verified in each animal by inspection under a stereomicroscope (Wild M3Z, Heerbrugg, Switzerland). Two VDR^Δ/Δ^ mice were excluded from the evaluation based on the arterial blood gas tensions and mean arterial blood pressure values, whereas one WT mouse was excluded because of incomplete occlusion of the carotid artery.

### 2.4. Measurement of Carotid Artery Blood Flow Using a Transit-Time Ultrasonic Flowmeter

For the continuous measurement of carotid artery blood flow, ultrasonic transit-time perivascular flow probe 0.5 PSB and TS420 flowmeter (Transonic System Inc, Ithaca, NY, USA) were used. The external carotid arteries were ligated in order to measure the blood flow to the brain as the flow probe was not applicable to the internal carotid arteries. Before starting the blood flow measurement, atipamezole was administered as described previously. The baseline blood flow and systemic blood pressure (via femoral artery cannula) were measured for 1 min before CAO. Thereafter, the left common carotid artery was occluded by placing a vessel clip on the artery distally to the flow probe. The contralateral carotid arterial blood flow and the systemic blood pressure were measured for 5 min after CAO, and the vascular conductance was calculated as follows: blood flow (mL/min)/mean arterial blood pressure (mmHg). The occlusion was considered successful if we measured zero flow in the ipsilateral carotid artery.

### 2.5. Evaluation of the Morphology of Pial Collaterals

For visualization of the cerebrocortical vasculature, mice were perfused transcardially with 10 mL heparinized (10 IU/mL) saline solution followed by the injection of 2 mL mixture of black inks (drawing ink (KOH-I-NOOR HARDTMUTH, České Budějovice, Czech Republic) and endorsing ink (INTERACTION-CONNECT, Gent, Belgium) and distilled water at a 6:1:6 ratio into the left cardiac ventricle under isoflurane (2%) anesthesia. Thereafter, the mice were decapitated, and the brains were removed and immersed immediately in a 4% formaldehyde solution for 24 h. Four WT and two VDR^Δ/Δ^ mice were excluded from the study because of unstained pial vessels, while six animals in each group were used for evaluation. Pictures of the dorsal surface of the brain were taken with a digital camera (Leica MC 190 HD, Leica Microsystems, Wetzlar, Germany) connected to a microscope (Leica M80, Leica Microsystems, Wetzlar, Germany). The morphological analysis of the leptomeningeal collaterals connecting the branches of the anterior cerebral artery (ACA) and middle cerebral artery (MCA) was performed using ImageJ software (Image J 1.5 NIH, Bethesda, MD, USA). For the calibration, a micrometer etalon was used. The number and the tortuosity index (the ratio of vessel curve length over the line distance between the two ends of the vessel) of the collaterals of both hemispheres were determined by a blinded investigator. The anastomotic points were identified as the half distance between the nearest branching points of the ACA and the MCA branches [22]. Adjacent anastomotic points were connected by the anastomotic line, and the distance between the anastomotic line and the midline at 4 mm caudally from the frontal pole of the brain (level of bregma) was measured [23,24].

### 2.6. Data Analysis

The normal distribution of datasets was checked with the Shapiro–Wilk test. If the normal distribution was verified, data were presented as the arithmetic mean and standard deviation and the *p*-values were determined by Student’s unpaired *t*-test or by two-way repeated measures ANOVA followed by Bonferroni post hoc test. If data were not normally distributed, they were presented as the median and interquartile range, for which the Mann–Whitney test was used to determine statistical significance. Statistical analysis and graph plotting were performed with GraphPad Prism software (v.6.07; GraphPad Software Inc., La Jolla, CA, USA) and *p* < 0.05 was considered as a statistically significant difference.

## 3. Results

### 3.1. Physiological Parameters

In order to examine whether deficiency of VDR signaling impacts general physiological parameters in mice, blood pressure, heart rate, and respiratory rate were recorded during the in vivo measurements. In addition, the weight of the body, heart, brain, and left ventricle, as well as the length of the tibia, the hematocrit level, and the plasma concentrations of ions (Ca^2+^, Na^+^, K^+^, Cl^−^) were measured at the end of each experiment. Functional inactivation of VDR resulted in decreased body weight and tibial length (Table 1). However, no changes in any other parameters including heart and brain weight were detected (Table 1), indicating that VDR inactivity results in reduced body weight probably due to altered skeletal development without any alterations in the heart and brain size. The plasma concentrations of calcium ion did not differ either between the groups which could be attributed to the rescue diet since it is able to normalize calcium homeostasis in VDR^Δ/Δ^ mice [21].

### 3.2. Effects of VDR Deficiency on the Regional CoBF Changes after CAO

**Figure 2:**
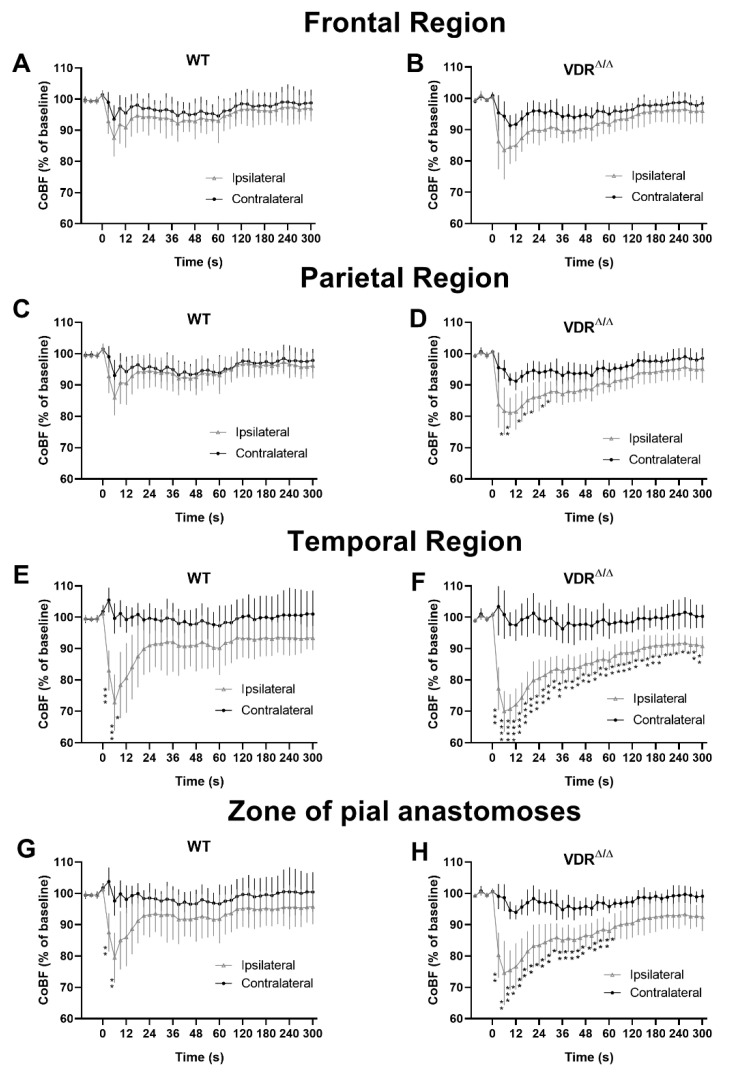
Regional cerebrocortical blood flow (CoBF) changes in WT (**A**,**C**,**E**,**G**) and VDR^Δ/Δ^ (**B**,**D**,**F**,**H**) mice following carotid artery occlusion (CAO). Zero time indicates the moment of the left carotid artery occlusion. Black circles and gray triangles represent the CoBF in the contralateral and ipsilateral hemispheres, respectively. CAO did not cause any changes in the CoBF of the ipsilateral frontal cortex as compared to the contralateral one (**A**) in WT or (**B**) VDR^Δ/Δ^ mice. (**C**) CAO did not induce any changes in the CoBF of the ipsilateral parietal cortex as compared to the contralateral one in WT mice, whereas (**D**) it resulted in a significant CoBF reduction in the acute phase in VDR^Δ/Δ^ mice. (**E**,**G**) The CoBF of the temporal cortex and the zone of pial anastomoses ipsilateral to CAO were reduced significantly only in the first few seconds after CAO in WT animals, whereas (**F**,**H**) a more prolonged CoBF reduction of both zones was determined in VDR^Δ/Δ^ mice. (* *p* < 0.05, ** *p* < 0.01, *** *p* < 0.001, **** *p* < 0.0001, *n* = 8 in both groups, two-way repeated measures ANOVA followed by Bonferroni post hoc test). Data are presented as mean ± SD.

First, CoBF changes were analyzed in four different cerebrocortical regions in order to examine the effect of CAO on regional CoBF in the two experimental groups. Qualitative assessment of the spatiotemporal pattern of CoBF reduction already indicated more pronounced changes in VDR deficiency both in terms of the extent and the duration of the hypoperfusion (Figure 1 and Video S1). Quantitatively, in the frontal region ipsilateral to CAO neither WT nor VDR^Δ/Δ^ mice showed significant CoBF reduction after CAO as compared to the contralateral side (Figure 2A,B) and similar results were obtained in the parietal region of WT animals (Figure 2C). In the parietal region of VDR^Δ/Δ^ mice, however, the CoBF reduced significantly in the acute phase after CAO and normalized thereafter (Figure 2D). More pronounced changes were observed in the temporal region: in WT animals, CAO resulted in pronounced but transient hypoperfusion in the ipsilateral temporal cortex, whereas in VDR^Δ/Δ^ mice CoBF remained significantly reduced during the whole measurement (Figure 2E,F). In the zone of pial anastomoses both WT and VDR^Δ/Δ^ mice showed transient hypoperfusion, although the CoBF normalized much earlier in WT than VDR^Δ/Δ^ animals (Figure 2G,H).

**Figure 3:**
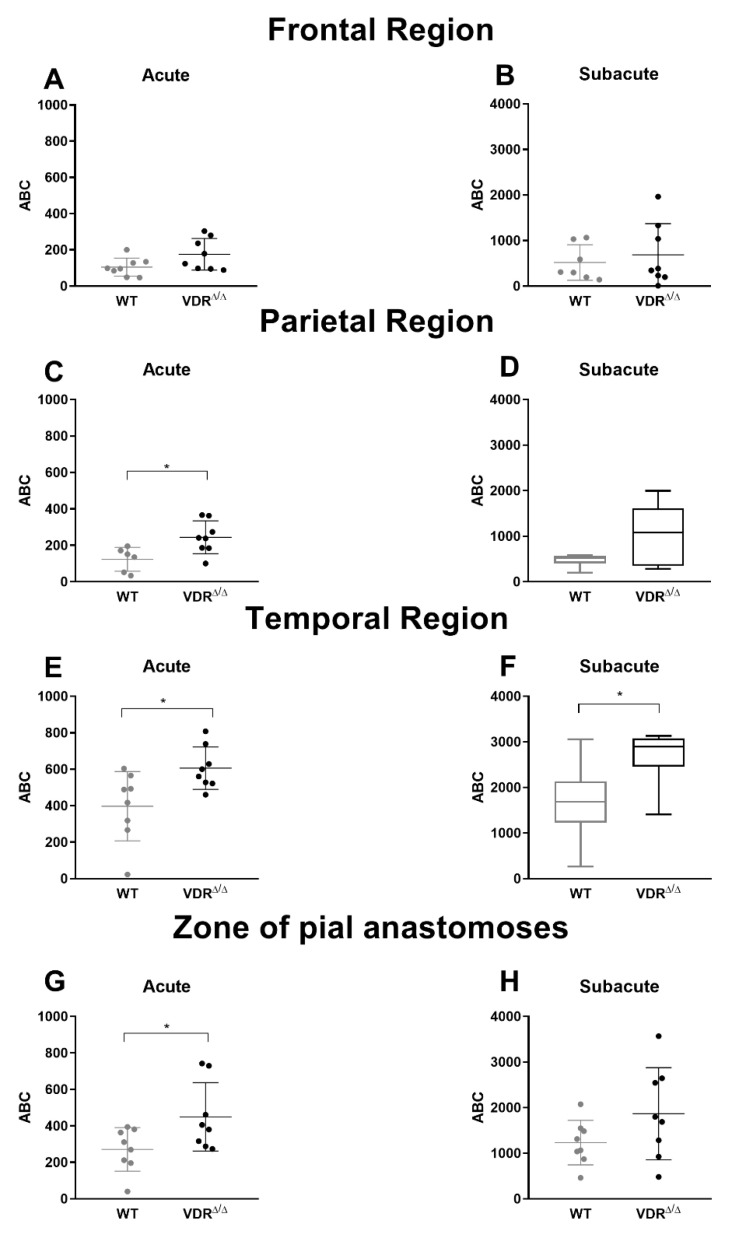
Differences in regional cerebrocortical blood flow (CoBF) induced by carotid artery occlusion (CAO) in VDR^Δ/Δ^ vs WT mice in the acute (0–30 s after CAO) and the subacute (30–300 s after CAO) phases of adaptation. The CoBF reductions induced directly by CAO were determined as the area between the curves (ABC) of CoBF reductions ipsilateral and contralateral to CAO. (**A**,**B**) In the frontal cortex, the ABC did not differ between the groups (*n* = 8 in both groups, Student’s unpaired *t*-test). (**C**) The ABC in the parietal cortex was increased in VDR^Δ/Δ^ mice as compared to WT animals in the acute phase (* *p* < 0.05, *n* = 8, Student’s unpaired *t*-test) indicating more severe hypoperfusion in the VDR^Δ/Δ^ mice, however, (**D**) this difference disappeared in the subacute phase (*n* = 8, Mann–Whitney test). (**E**,**F**) VDR inactivity resulted in increased ABC (i.e., decreased CoBF) in the ipsilateral temporal cortex both in the acute (**E**, * *p* < 0.05, *n* = 8, Student’s unpaired *t*-test) and in the subacute phase (**F**, * *p* < 0.05, *n* = 8, Mann–Whitney test). (**G**) ABC was increased in the zone of pial anastomoses of VDR^Δ/Δ^ mice as compared to WT animals in the acute phase (* *p* < 0.05, *n* = 8, Student’s unpaired *t*-test) but (**H**) not in the subacute phase (*n* = 8, Student’s unpaired *t*-test). Normally distributed data are presented as a scatter dot plot with mean ± SD, whereas not normally distributed data are shown as a box and whisker plot with median and interquartile range.

**Figure 4:**
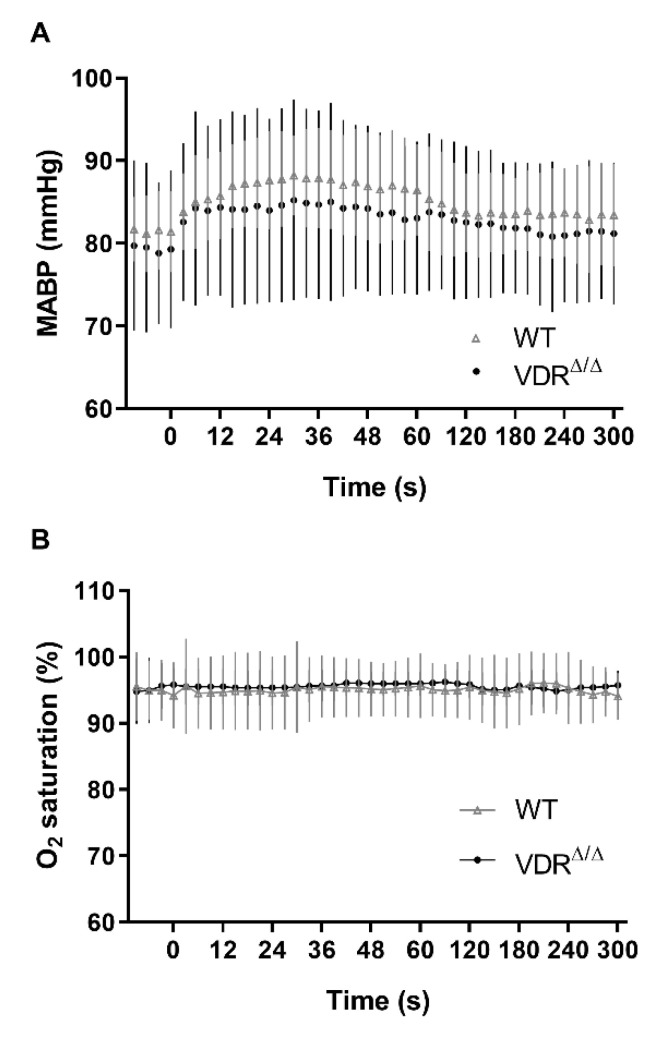
Physiological parameters of mice during the cerebrocortical blood flow measurement. (**A**) The mean arterial blood pressure (MABP) and (**B**) the O_2_ saturation were within the physiological range throughout the experiments, and carotid artery occlusion induced only a minor increase in the MABP. Zero time indicates the moment of the left carotid artery occlusion. Neither parameters were different between the groups (*n* = 8 in both groups, two-way repeated measures ANOVA followed by Bonferroni post hoc test). Data are presented as mean ± SD.

Next, we aimed to quantify the differences in regional CoBF changes induced by CAO in VDR^Δ/Δ^ vs. WT mice. In order to pinpoint the direct effect of CAO unmasked by CoBF alterations related to potential fluctuations of systemic physiological parameters (e.g., arterial blood pressure or blood gas values), we determined the area between the CoBF curves (expressed as the percentage of the baseline) of the hemispheres ipsilateral and contralateral to CAO for each mouse. Furthermore, we differentiated between the acute (0–30 s after CAO) and subacute (30–300 s after CAO) phases of CoBF changes as autoregulation may involve different mechanisms with time [17]. VDR inactivity caused a more pronounced decrease of CoBF in the parietal region and in the zone of pial anastomoses in the acute phase (Figure 3C,G) indicating an impaired cerebral vasoregulation; whereas during the subacute phase, this difference disappeared (Figure 3D,H). In contrast, the CoBF reduction was more pronounced and prolonged in the temporal cortex of VDR^Δ/Δ^ mice—as it was increased both in the acute and subacute phases—as compared to the WT animals (Figure 3E,F), indicating a more severe vasoregulatory dysfunction in the temporal cortex of VDR^Δ/Δ^ mice. We could exclude the possibility that any differences in the systemic arterial blood pressure or the arterial blood gas tensions would have caused the aforementioned differences between the groups as neither the mean arterial pressure (Figure 4A) nor the arterial blood gas and acid-base parameters (Table 2) were different between the WT and VDR^Δ/Δ^ mice. In addition, CAO did not alter the arterial O_2_ saturation (Figure 4B) and induced only a minor transient increase in the mean arterial blood pressure in both groups (Figure 4A).

### 3.3. Effects of VDR Deficiency on the Extracranial Collateral Circulation 

**Figure 5:**
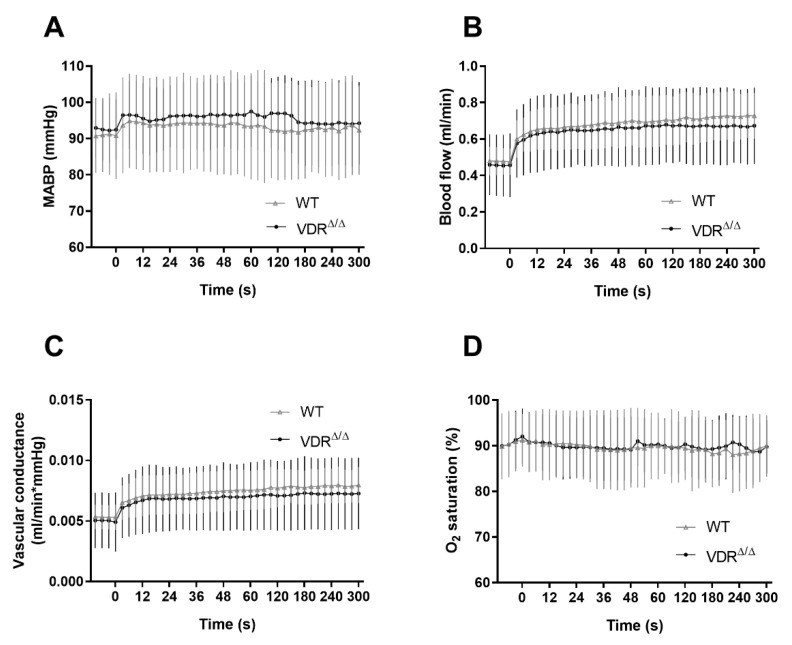
Changes of the systemic blood pressure, contralateral carotid arterial blood flow, and vascular conductance as well as arterial O_2_ saturation after unilateral carotid artery occlusion (CAO). (**A**) CAO caused a minor increase in the mean arterial blood pressure (MABP) and (**B**) a more pronounced increase in the contralateral carotid artery blood flow, resulting (**C**) in increased vascular conductance of the contralateral carotid artery. (**D**) The O_2_ saturation was not affected by CAO. Zero time indicates the moment of the left carotid artery occlusion. Neither parameters were different between the groups (*n* = 6 in both groups, two-way repeated measures ANOVA followed by Bonferroni post hoc test). Data are presented as mean ± SD.

A possible explanation for the altered adaptation of the cerebrocortical circulation in VDR deficiency could be the diminished compensatory blood flow elevation of the contralateral carotid artery, the main alternative route of blood supply to the brain after CAO. In order to test this hypothesis, we determined changes in blood flow and vascular conductance of the contralateral carotid artery in WT and VDR^Δ/Δ^ mice. The blood flow increased significantly after CAO without any major changes in the blood pressure in WT mice, therefore, the vascular conductance was also enhanced (Figure 5A–C). Since these changes were not accompanied by any alteration of the arterial O_2_ saturation (Figure 5D), the increase of the oxygen supply to the brain via the contralateral carotid artery was proportional to the blood flow changes. However, none of these parameters was affected by VDR deficiency (Figure 5) indicating an unchanged adaptation of the contralateral carotid artery to CAO. Therefore, we rejected the hypothesis that the altered recovery of CoBF would be related to the diminished extracranial collateral circulation in VDR^Δ/Δ^ mice.

### 3.4. Effects of VDR Deficiency on the Intracranial Collateral Circulation

**Figure 6:**
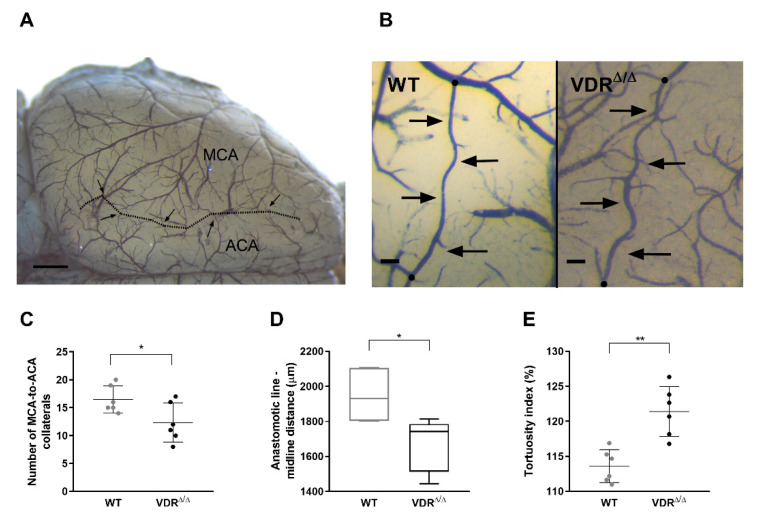
Evaluation of the morphology of intracranial collaterals. (**A**) Representative image of the dorsal surface of the brain infused with the mixture of black inks. The dotted line depicts the anastomotic line, whereas arrows denote the collaterals between the branches of the middle cerebral artery (MCA) and anterior cerebral artery (ACA). Scale bar represents 1000 μm. (**B**) Representative images indicating the increased tortuosity of the collaterals of VDR^Δ/Δ^ mice (right) as compared to WT mice (left). Arrows show the path of one MCA-to-ACA collateral, whereas black dots denote the end points of the collateral. Scale bars represent 100 μm. (**C**) Functional inactivity of VDR decreased the number of MCA-to-ACA collaterals (* *p* < 0.05, *n* = 6 in both groups, Student’s unpaired *t*-test). (**D**) The anastomotic line was closer to the midline at 4 mm posterior from the frontal pole (level of bregma) in VDR^Δ/Δ^ mice as compared to WT animals (* *p* < 0.05, *n* = 6, Mann–Whitney test). (**E**) The tortuosity index was increased in VDR^Δ/Δ^ mice as compared to WT animals (** *p* < 0.001, *n* = 6, Student’s unpaired *t*-test). Normally distributed data are presented as scatter dot plots, whereas not normally distributed data are shown as box and whisker plots; *n* indicates the number of brains analyzed.

The efficiency of pial collateral circulation is one of the major determinants of the favorable cerebrovascular adaptation to occlusion of the major cerebral arteries [25]. Therefore, we hypothesized that unfavorable alterations of the leptomeningeal anastomoses of the VDR-deficient mice may account for the diminished recovery of the CoBF after CAO. In order to test this hypothesis, the number and the tortuosity of collaterals between the cortical branches of MCA and ACA were determined (Figure 6A). In VDR^Δ/Δ^ mice a significant reduction was observed on the one hand in the number of pial MCA-to-ACA collaterals (Figure 6C); on the other hand, a significantly increased tortuosity of these collateral vessels was observed (Figure 6B,E) indicating an impaired development of leptomeningeal anastomoses. In addition, the distance of the anastomotic line from the midline was measured in order to distinguish the cortical territories supplied by the MCA and ACA. Interestingly, the anastomotic line was closer to the midline in VDR^Δ/Δ^ mice as compared to WT animals (Figure 6D) indicating that the territory supplied by the MCA was increased at the expense of the territory of ACA. All these alterations have a negative impact on the capacity of leptomeningeal collaterals to contribute to the adaptation of the cerebrocortical circulation to CAO and can explain the more pronounced drop and delayed recovery of the CoBF in the temporal cortex of VDR^Δ/Δ^ mice.

## 4. Discussion

In the present study, we observed morphological and functional alterations in the cerebrovascular system of mice carrying functionally inactive VDR (a mouse model of HVDRR [21]). Ablation of the VDR in mice has been widely used for investigating the role of vitamin D signaling in several physiological functions [26]. VDR-deficient mice in our present study showed certain phenotype such as lower body weight and shortened tibial length. On the contrary, we did not observe any major alterations in the cardiovascular system. The present finding confirms previous reports [21,27,28,29], although there is a discrepancy regarding the heart weight/body weight ratio. This could be because either older mice were examined or the animals did not receive the rescue diet for normalizing the plasma Ca^2+^ levels [21]. In support of this concept, Andrukhova et al. found that 9-month-old VDR ablated mice on rescue diet had increased heart weight/body weight ratio, however, they did not observe this difference in younger animals [27]. Similarly, other studies reported increased heart weight/body weight ratio of 12-month-old VDR-deficient mice [30,31] or younger animals not receiving the rescue diet [32].

Vitamin D insufficiency appears to be associated with cerebrovascular diseases including ischemic stroke [10,11], however, it is uncertain whether vitamin D signaling is involved in the adaptation of cerebral circulation to ischemia or not. In order to elucidate the role of vitamin D, we examined the cerebrovascular adaptation to unilateral CAO in VDR ablated mice. It was reported previously that unilateral CAO induces a rapid reduction in the CoBF of the ipsilateral hemisphere of mice, but within 30 s, the CoBF starts to increase and returns close to the baseline level [17]. However, functional inactivation of the nuclear VDR appears to impede the rapid recovery of CoBF following CAO since we determined more pronounced CoBF reductions in the ipsilateral hemisphere of VDR^Δ/Δ^ mice as compared to WT animals, indicating an impaired vasoregulation in VDR deficiency. 

Occlusion of a major artery supplying the brain could compromise the cerebral circulation, however, after unilateral CAO the extracranial collateral circulation (i.e., the contralateral carotid and the vertebral arteries) can provide an alternative route for blood supply to the brain [25]. In mice, the role of the contralateral artery could be especially important since the posterior communicating arteries are less developed [33]. Efficient blood supply from the contralateral side in mice is provided due to the azygous anterior cerebral artery (AACA). The AACA supplies the frontal-parietal regions of both hemispheres, and it raises from the fusion of ACAs of both sides [33]. Therefore, both carotid arteries can provide the blood supply of the frontal-parietal cortices which may improve cerebrovascular adaptation to CAO [17,33]. Vitamin D deficiency, however, has been reported to impair flow-mediated vasodilation [34] possibly due to diminishing nitric-oxide bioavailability [27,35,36,37] and disinhibiting the formation of reactive oxygen species [38,39,40], which would imply an impairment of the contralateral carotid arterial blood flow increase following CAO. Surprisingly, however, VDR inactivity did not affect the increase in blood flow and vascular conductance of the contralateral carotid artery indicating that the compromised capacity of VDR^Δ/Δ^ mice for cerebrovascular adaptation is independent of the extracranial collateral circulation. In addition, we could exclude the possibility that any changes in systemic arterial blood pressure or those of the blood gas parameters would be responsible for the more severe and prolonged hypoperfusion of VDR^Δ/Δ^ mice since we found no differences in those parameters between the WT and VDR^Δ/Δ^ animals.

The temporal cortex appears to be affected most severely by CAO [17] but the collateral circulation through pial anastomoses could attenuate its ischemia [17,41,42,43]. Our results indicate that this compensatory mechanism did not work sufficiently in the absence of vitamin D signaling because the CoBF reduction in the temporal cortex was more severe and prolonged (i.e., the recovery was slower in VDR^Δ/Δ^ mice as compared to the WT animals). To evaluate the capacity of the intracranial collateral circulation to compensate for the blood loss of the temporal cortex, we examined the pial collaterals between the cortical branches of the MCA and the ACA. The abundance of leptomeningeal anastomoses among the terminal branches of the three large cerebral arteries is the highest between the MCA and the ACA, and these collaterals might be particularly important for the blood flow redistribution between the more severely affected temporal cortex and the less severely impacted frontal and parietal regions after CAO [17,44]. In the anastomoses, the blood can flow in both directions depending on the hemodynamic status and metabolic needs of the connected territories, therefore, they can improve the blood supply of the ischemic region [45]. The extent of the pial collateral network appears to be inversely associated with the cortical infarct size [41,46], therefore, the decreased number of anastomoses in VDR^Δ/Δ^ mice could exacerbate the consequences of ischemic stroke. The decreased collateral number was accompanied by increased tortuosity of anastomoses in VDR^Δ/Δ^ mice, which further compromises the collateral circulation. The increased vascular tortuosity implies the development of local turbulence and may cause abnormal shear stress in the vessel wall, resulting in impaired flow-induced vasodilation, which may ultimately lead to the generation of atherosclerosis [47]. In addition to the differences in collateral density and morphology, the size of the MCA and ACA tree (i.e., the cerebral territory supplied by the MCA and the ACA) impacts the outcome of stroke [48]. Functional inactivation of VDR increased the territory of MCA at the expense of the territory of ACA, which might further impair the compensatory capacity since a larger territory has to be supplied by the less developed collateral network.

Endothelial dysfunction [19,27,35] or diminished vascular endothelial growth factor (VEGF) expression during the embryogenic life [49] could be responsible for the impaired collateral development in VDR deficiency since both VEGF and endothelial nitric-oxide synthase deficiency appears to disturb collateral development [50,51,52]. A limitation of the present study is that we were unable to determine the exact diameter of collaterals because our method used for the visualization of cerebral vessels could not provide accurate measurement of diameters. However, alterations in collateral vessel diameter could also impact the blood flow redistribution capacity of anastomoses [53,54]. Nevertheless, our results indicate that vitamin D plays an important role in collateral development and in turn, the absence of vitamin D, especially in the prenatal or perinatal period [55], could be responsible for the prolonged CoBF reduction in the temporal cortex.

Unlike the temporal region, the frontal and parietal cortex are better protected from unilateral CAO, especially in mice, because of the common blood supply of the two hemispheres by the AACA [33]. Despite the lower vulnerability of the frontal-parietal region, the CoBF decreased significantly and remained reduced in the acute phase (i.e., until 30 s after CAO) in the parietal cortex in VDR^Δ/Δ^ mice implying an impaired vasoregulation. We presume that the collateral pathway represented by the large vessels of the Willis circle is impaired in VDR inactivity since we reported previously that vitamin D deficiency induces morphological alterations and impaired endothelium-mediated vasodilation in anterior cerebral arteries [19]. Therefore, the compromised flow-induced vasodilatory capacity of the vessels of the Willis circle could be responsible for the insufficient blood flow redistribution in the acute phase (0–30 s after CAO) and in turn for the lack of immediate adaptation to CAO in VDR deficiency. On the contrary, in the subacute phase (30–300 s after CAO), the impaired development of pial collaterals in VDR deficiency may hinder the draining effect between the temporal and frontal-parietal region via pial anastomoses; therefore, the most severely affected temporal cortex could not ”steal” blood from the less severely affected regions.

Our results indicate that lack of vitamin D signaling may impair the cerebrovascular adaptation to ischemia and in turn, may worsen the outcome of stroke. With this notion, vitamin D deficiency has been reported to increase the infarction volume, exacerbate behavioral impairment, and compromise the blood–brain barrier after cerebrovascular occlusion [56,57]. However, Evans et al. found that vitamin D deficiency had no effect on the extent of brain injury [58]. The discrepancy could be explained by the different onset and type of vitamin D deficiency. In the present study, mice carrying a functionally inactive VDR were exposed to the absence of vitamin D signaling already in the prenatal period, which appears to lead to more severe vulnerability to cardiovascular diseases than vitamin D deficiency developing in adult life [59,60]. Accordingly, developmental vitamin D deficiency was reported to alter neuronal growth and differentiation, and it is associated with impaired development of the dopaminergic system [61] as well as with the risk of schizophrenia [62,63]. Furthermore, vitamin D deficiency appears to be linked to autism, Parkinson’s disease, cognitive impairments, and depression [62,63]. The beneficial role of vitamin D in the brain could be attributed—at least partly—to its neuroprotective effect [26], since vitamin D has been reported to modulate the expression of neurotrophic factors, ion channels, and inflammatory mediators [61]. Therefore, besides compromising cerebrovascular adaptation, vitamin D deficiency can also worsen the outcome of ischemic stroke by directly impairing neuronal functions.

## 5. Conclusions

The present study demonstrates the harmful effects of functional inactivation of VDR on the cerebrovascular adaptation to unilateral CAO. Surprisingly, the extracranial collateral circulation was not compromised by VDR deficiency. Therefore, the impaired cerebrovascular adaptation of VDR^Δ/Δ^ mice could be attributed to diminished intracranial collateral circulation. The more pronounced reductions of CoBF in VDR^Δ/Δ^ mice during the acute phase after CAO indicate an insufficient vasoregulation and blood flow redistribution in the Willis circle, whereas the impaired development of leptomeningeal anastomoses might be responsible for the diminished blood flow recovery of the most severely affected temporal cortex. These results emphasize the importance of vitamin D signaling in normal cerebrovascular development and the prevention of cerebrovascular disorders.

## Figures and Tables

**Table 1:** Physiological parameters.

Parameter	WT	VDR^Δ/Δ^
Body weight (g)	30.0 ± 2.8	27.3 ± 2.4 **
Tibial length (cm)	1.80 (1.80–1.82)	1.70 (1.60–1.70) ****
Heart weight (g)	0.19 ± 0.03	0.17 ± 0.03
Heart weight/body weight (%)	0.60 ± 0.08	0.62 ± 0.11
Left ventricle weight (g)	0.12 (0.11–0.12)	0.10 (0.09–0.11)
Brain weight (g)	0.47 (0.44–0.48)	0.45 (0.44–0.46)
Blood pressure (mmHg)	84.09 ± 8.38	82.97 ± 9.60
Heart rate (1/min)	364.8 (310.3–423.3)	351.6 (320.7–409.8)
Respiratory rate (1/min)	59.56 (52.99–82.40)	62.56 (54.42–78.91)
Hematocrit (%)	42.63 ± 2.26	43.57 ± 1.51
cNa^+^ (mmol/L)	156.5 ± 3.67	156.7 ± 3.64
cK^+^ (mmol/L)	4.30 (3.67–4.46)	4.02 (3.62–4.22)
cCa^2+^ (mmol/L)	1.29 ± 0.09	1.24 ± 0.07
cCl^−^ (mmol/L)	113.4 ± 2.50	115.5 ± 4.96

Functional inactivation of the VDR caused a decrease in the body weight and the tibial length of mice (** *p* < 0.01, Student’s unpaired *t*-test; **** *p* < 0.0001, Mann–Whitney test). There were no differences in any other physiological parameters between the WT and VDR^Δ/Δ^ animals. Data are presented as mean ± SD or median and interquartile range; *n* = 8 for the hematocrit level and the plasma concentrations of ions, whereas *n* = 14 for any other parameters.

**Table 2:** Arterial blood gas and acid-base parameters.

Parameter	WT	VDR^Δ/Δ^
pH	7.31 ± 0.10	7.29 ± 0.04
pCO_2_ (mmHg)	41.16 ± 9.13	42.51 ± 6.18
pO_2_ (mmHg)	93.5 (89.0–103.09)	109.0 (85.5–112.8)
Standard base excess (mmol/L)	−5.08 ± 2.94	−5.66 ± 2.81
O_2_ saturation (%)	96.10 (94.95–97.58)	97.60 (93.40–97.98)

Data are presented as mean ± SD or median and interquartile range, *n* = 8 in both groups.

## Supplementary Materials

The following are available online at https://www.mdpi.com/2073-4409/9/6/1457/s1, Video S1: Cerebrocortical blood flow (CoBF) reductions after left carotid artery occlusion (CAO) in a mouse carrying functionally inactive vitamin D receptor (VDR^Δ/Δ^) and its wild-type (WT) littermate.
