# Supplementary material for: Ablation of Vitamin D Signaling Compromises Cerebrovascular Adaptation to Carotid Artery Occlusion in Mice
Source: Cells. 2020 Jun 12;9(6):1457. doi: 10.3390/cells9061457 (PMC7349396; doi:10.3390/cells9061457)
Supplement: Supplementary file 1 [file cells-09-01457-s001.zip › cells-831008 supplementary final/cells-831008 supplementary .docx]

**Video S1.** Cerebrocortical blood flow (CoBF) reductions after left carotid artery occlusion (CAO) in a mouse carrying functionally inactive vitamin D receptor (VDR^Δ/Δ^) and in its wild-type (WT) littermate. The localization of the regions of interest for CoBF measurements is depicted. The video shows five-fold acceleration the CoBF changes for 30 s preceding CAO and until 180 s after CAO. The decrease in CoBF ipsilateral to carotid artery occlusion was more pronounced in VDR^Δ/Δ^ mice as compared to WT littermates with the most sustained reductions in the temporal cortex.
